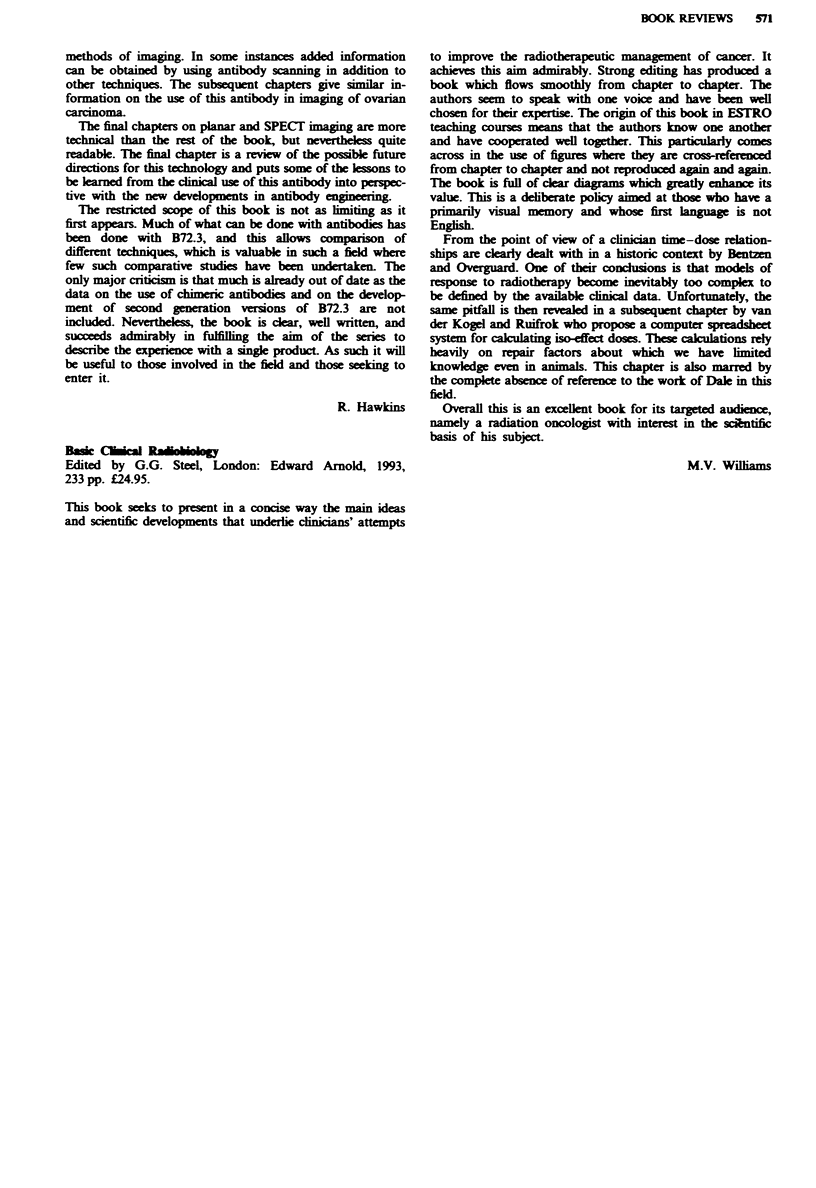# Basic clinical radiobiology

**Published:** 1994-09

**Authors:** M. V. Williams


**Basic Clinical Radiobiology**

Edited by G.G. Steel, London: Edward Arnold, 1993, 233 pp. £24.95.

This book seeks to present in a concise way the main ideas and scientific developments that underlie clinicians' attempts to improve the radiotherapeutic management of cancer. It achieves this aim admirably. Strong editing has produced a book which flows smoothly from chapter to chapter. The authors seem to speak with one voice and have been well chosen for their expertise. The origin of this book in ESTRO teaching courses means that the authors know one another and have cooperated well together. This particularly comes across in the use of figures where they are cross-referenced from chapter to chapter and not reproduced again and again. The book is full of clear diagrams which greatly enhance its value. This is a deliberate policy aimed at those who have a primarily visual memory and whose first language is not English.

From the point of view of a clinician time–dose relationships are clearly dealt with in a historic context by Bentzen and Overguard. One of their conclusions is that models of response to radiotherapy become inevitably too complex to be defined by the available clinical data. Unfortunately, the same pitfall is then revealed in a subsequent chapter by van der Kogel and Ruifrok who propose a computer spreadsheet system for calculating iso-effect doses. These calculations rely heavily on repair factors about which we have limited knowledge even in animals. This chapter is also marred by the complete absence of reference to the work of Dale in this field.

Overall this is an excellent book for its targeted audience, namely a radiation oncologist with interest in the scientific basis of his subject.

M.V. Williams